# Disseminated Superficial Non-actinic Porokeratosis: A Consequence of Post-traumatic Immunosuppression

**DOI:** 10.7759/cureus.73218

**Published:** 2024-11-07

**Authors:** Liliana G Popa, Teodora Cristiana Gradinaru, Calin Giurcaneanu, Irina Tudose, Cristina Iolanda Vivisenco, Cristina Beiu

**Affiliations:** 1 Dermatology, Elias Emergency University Hospital, Carol Davila University of Medicine and Pharmacy, Bucharest, ROU; 2 Dermatology, Elias Emergency University Hospital, Bucharest, ROU; 3 Biochemistry, Carol Davila University of Medicine and Pharmacy, Bucharest, ROU; 4 Pathology, Elias Emergency University Hospital, Bucharest, ROU; 5 Pediatrics, Grigore Alexandrescu Clinical Emergency Hospital, Bucharest, ROU; 6 Oncologic Dermatology, Elias Emergency University Hospital, Carol Davila University of Medicine and Pharmacy, Bucharest, ROU

**Keywords:** immunosuppression, major surgery, non-actinic, porokeratosis, trauma

## Abstract

Disseminated superficial porokeratosis (DSP) is a very uncommon dermatologic condition of unknown etiology, characterized by the clonal proliferation of atypical keratinocytes associated with aberrant keratinocyte differentiation. These lead to the development of the specific cornoid lamella that separates atypical from normal keratinocytes. DSP is most frequently encountered in immunosuppressed patients. It has been described in patients receiving immunosuppressive treatments, organ transplantation and in patients diagnosed with human immunodeficiency virus, hepatitis B and hepatitis C virus infection. We present the case of a 78-year-old patient who developed disseminated non-actinic after multiple trauma and major orthopedic surgery. To our knowledge, this is the first case of DSP occurring as a consequence of post-traumatic immunosuppression reported in the medical literature. We discuss the pathogenic mechanisms, as well as the optimal diagnostic and therapeutic approach in such cases.

## Introduction

Porokeratosis is a rare dermatologic disorder of unknown etiology, which comprises numerous clinical variants that can be both acquired and inherited. Traditionally classified as a keratinization disorder, a recent study highlights the possibility of reframing porokeratosis as a genodermatosis, in fact, one of the most common ones [1]. The involvement of mevalonate pathway genes has been outlined for many clinical variants of this disease [2,3].

First described in 1875 by Neumann [4], it was not until 1893 that Mibelli introduced the term "porokeratosis” (from the Greek *poor* (pore) and *keratosis* (horny thickening)) [5]. Although disseminated superficial porokeratosis was first characterized by Respighi in 1893 [6], it was only denominated in 1937 by Andrews [7]. Thirty years later, Chernoski and Freeman proposed “disseminated superficial actinic porokeratosis” (DSAP) as a distinctive entity [8].

Porokeratosis is a very uncommon disease that predominantly affects fair-skinned individuals and may occur at any age. While porokeratosis of Mibelli and porokeratosis palmaris et plantaris disseminata (PPPD) show a predilection for the male gender, DSAP is twice more common among women [9]. Even if the disease was described more than 100 years ago, its etiology and specific pathogenic mechanisms remain unclear. However, the role of a series of predisposing factors has been proven.

Genetic mutations in the mevalonate pathway have been linked with the group of porokeratosis diseases. Examples of mutated genes identified within the porokeratosis cluster include: mevalonate kinase gene, phosphomevalonate kinase gene, mevalonate (diphospho) decarboxylase gene, and farnesyl diphosphate synthase gene [2].

DSAP is a classic example of chronic photoexposure-induced dermatosis. As the term clearly suggests, in this clinical variant, the specific lesions appear on sun-exposed skin areas [10]. In their case report, Kawara et al. outlined the involvement of repeated exposure to narrowband ultraviolet B (NB-UVB) used for psoriasis treatment in the appearance of DSAP [11]. A systematic review of 25 studies on DSAP concluded that six patients developed the disease after undergoing different types of phototherapy (psoralen UVA, UVB, NB-UVB) [12]. 

Immunosuppression is another factor associated with the development of DSAP. Lu et al. reviewed the medical literature and identified six reports of patients who developed eruptive porokeratosis of the trunk and extremities, disseminated superficial porokeratosis (DSP) or DSAP following biological treatment with adalimumab, etanercept, exemestane, nivolumab, pembrolizumab, or trastuzumab [12]. Organ transplantation, especially renal transplant [13], but also heart transplant [14] and autologous bone marrow transplantation [15] have also been linked to the development of porokeratosis. Additionally, cases of genitogluteal porokeratosis [16] and DSP associated with human immunodeficiency virus (HIV) infection have been reported [17]. Moreover, hepatitis C virus (HCV) infection seems to be a predisposing factor for porokeratosis given the immunosuppression that ensues, as suggested by Mizukawa and Shiohara in a study that presented three cases of different types of porokeratosis occurring in hepatitis C patients [18]. In 2000, Kono et al. highlighted the possibility of DSP arising as a paraneoplastic skin sign in HCV-associated hepatocellular carcinoma [19]. A case of hyperkeratotic porokeratosis in a patient diagnosed with hepatitis B virus (HBV), hepatitis C virus (HCV) and human immunodeficiency virus (HIV) infection and treated with immunosuppressive therapy for psoriatic arthritis was also reported [20].

Other less explored factors have been associated with the occurrence of DSP. Nakamura et al. discussed the interesting case of a patient with sudden onset of DSP associated with severe diabetes mellitus due to anti-insulin antibodies [21].

Thus, porokeratosis is considered a multifactorial disease, characterized by the clonal proliferation of atypical keratinocytes associated with aberrant keratinocyte differentiation [22]. These processes generate the specific cornoid lamella that separates atypical from normal keratinocytes [23,24].

We present the case of a 78-year-old patient diagnosed with disseminated non-actinic porokeratosis occurring as a consequence of post-traumatic immunosuppression and discuss the pathogenic mechanisms, as well as the optimal diagnostic and therapeutic approach in such cases.

## Case presentation

**Figure 1:**
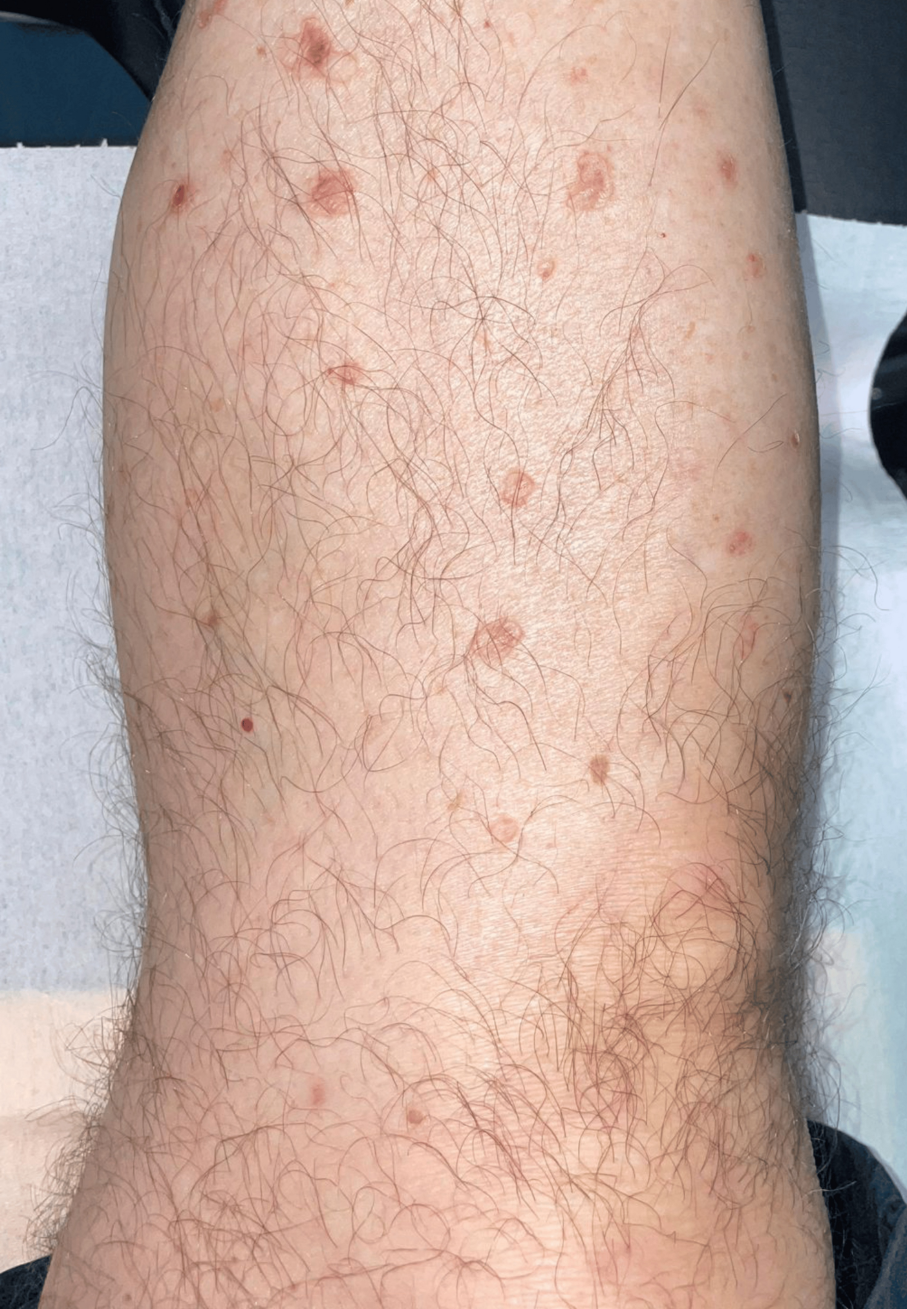
Clinical manifestations Annular macules displaying a hypopigmented, slightly atrophic center and a raised, scaly border disseminated on the lower limbs

**Figure 2:**
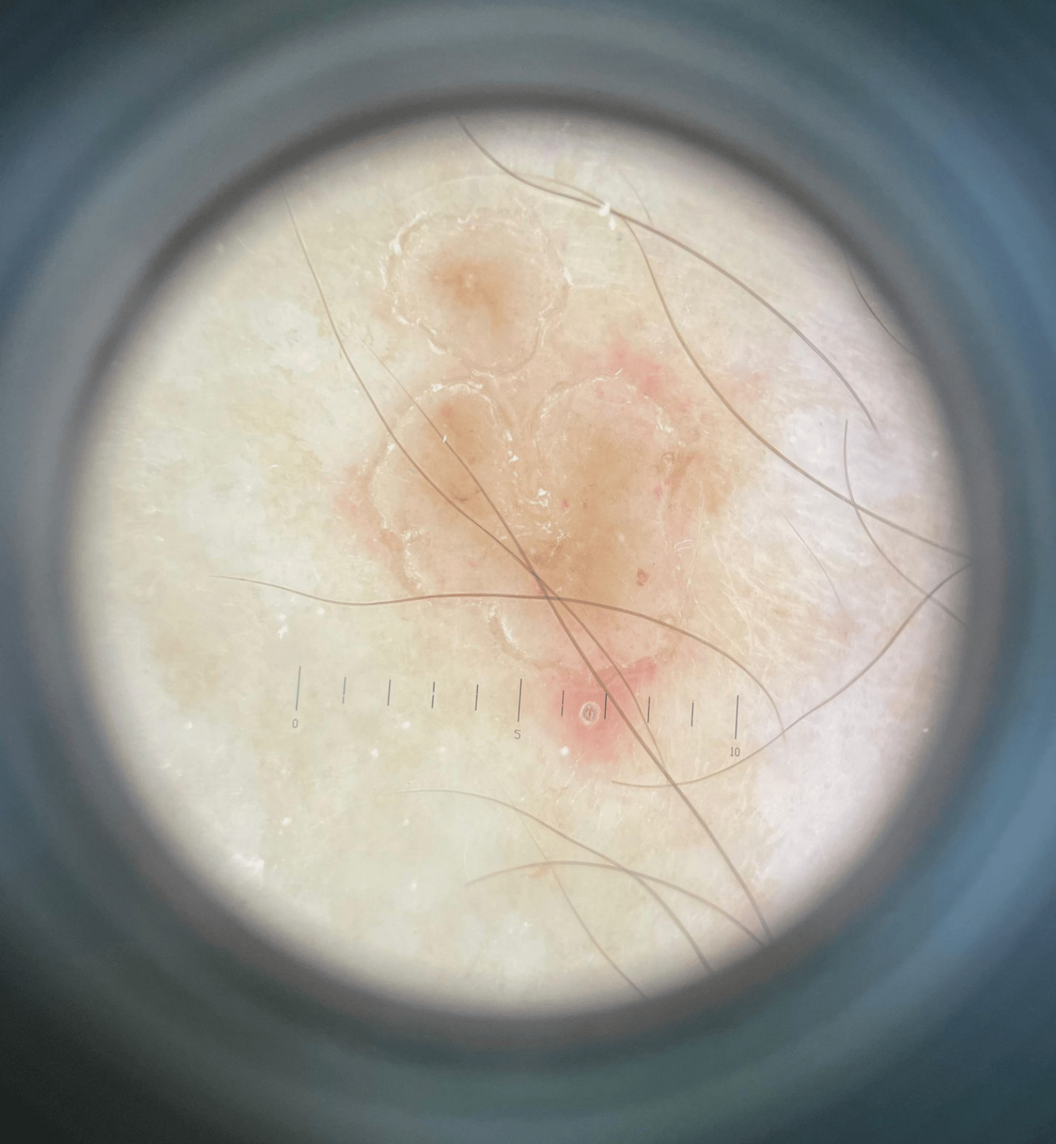
Dermoscopy Light brown lesions with a slightly raised, scaly border

A 78-year-old male patient was referred to our clinic for an asymptomatic rash consisting of annular macules displaying a hypopigmented, slightly atrophic center and a raised, scaly border disseminated on the trunk, upper and lower limbs (Figure 1). Dermoscopy revealed light brown lesions with a slightly raised, scaly border (Figure 2). The face, palmar and plantar surfaces were uninvolved. The patient first noticed the skin lesions a month previously, a few weeks after being hospitalized for the surgical treatment of multiple fractures following a car accident. The lesions slowly extended over the mentioned body areas. The patient suffered from arterial hypertension and was undergoing chronic antihypertensive treatment. He reported no other relevant personal or family medical history. 

**Figure 3:**
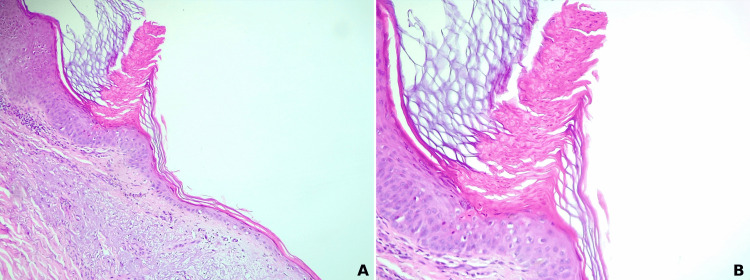
Hematoxylin-eosin stain Hematoxylin-eosin stain showing acanthosis, hyperkeratosis, the presence of a cornoid lamella composed of several layers of parakeratosis with a vertical disposition, with subjacent areas of hypogranulosis, few dyskeratotic epidermal cells, capillary ectasia and a chronic perivascular lymphocytic inflammatory infiltrate in the papillary dermis (A: 100x); magnified image of the parakeratotic column located at the periphery of the lesion with subjacent focal  hypogranulosis and vacuolated, dyskeratotic keratinocytes  (B: 200x).

A punch biopsy was performed from the hyperkeratotic ridge of one of the skin lesions and the histopathologic examination confirmed the clinical suspicion of porokeratosis, showing acanthosis, hyperkeratosis, the presence of a cornoid lamella composed of several layers of parakeratosis with a vertical disposition, with subjacent areas of hypogranulosis, few dyskeratotic epidermal cells, capillary ectasia and a chronic perivascular lymphocytic inflammatory infiltrate in the papillary dermis (Figure 3).

Considering the skin lesions developed during the winter months and were located in areas not exposed to sunlight, the diagnosis of DSP was established. Laboratory tests ruled out viral infections, including HBV, HCV, and HIV infections. Tumor markers were within normal limits. The thoracic x-ray and abdominal and pelvic ultrasound did not reveal pathologic findings.

The patient was reassured regarding the benign nature of the skin conditions. He refused further treatments and opted for the use of bland emollients. Three months later, the patient returned for a follow-up visit. The skin examination only revealed xerosis, the lesions having completely resolved.

## Discussion

Depending on the extension of the lesions, porokeratosis encompasses localized forms (porokeratosis of Mibelli, genital porokeratosis - ptychotropica, linear porokeratosis, giant porokeratosis, solar facial porokeratosis) and generalized forms (DSAP, DSP, eruptive bullous disseminated porokeratosis, pruriginous porokeratosis, follicular porokeratosis, palmo-plantar-disseminated porokeratosis) [25]. The real delimitation of clinical variants sometimes poses difficulties as the clinical presentation can be confusing or overlapping.

DSAP is the most commonly encountered among all types of porokeratosis [26]. The lesions involve sun-exposed areas and are worsened by photoexposure. On the other hand, DSP is not related to photoexposure. It affects both sun-exposed and unexposed areas (including the trunk, inguinal area, palms, and soles) and may rarely involve the oral mucosa [27] and genitalia [28]. Sometimes, it may be challenging to differentiate DSAP from DSP [29]. DSP is more frequently seen in immunosuppressed patients and children [30]. The development of DSP in our patient was most probably a consequence of post-traumatic immunosuppression.

In 1991, Bone et al. first described the post-traumatic systemic inflammatory response syndrome (SIRS), which includes fever, tachycardia, tachypnea, and leukocytosis/lekocytopenia [31]. Five years later, Bone refined the concept by reckoning a subsequent phenomenon termed “compensatory anti-inflammatory response syndrome (CARS)”, defined by altered function of antigen-presenting cells and macrophages, increased dendritic cell and T cell apoptosis, and polarization of the immune response to a Th2 phenotype [32]. Danger-associated molecular patterns, cytokines, especially interleukin (IL)-1β, IL-4, IL-6, IL-8, IL-10, tumor necrosis factor (TNF)-α chemokines, such as IL-8 and monocyte chemoattractant protein (MCP)-1 and other soluble mediators, like prostaglandin E2 (PGE2) and complement fragments C3 and C5 released at the site of injury greatly impact the innate and adaptive immune responses [33]. Cytokine production is directly proportional to trauma severity [34,35]. Due to the close mutual influences between the endocrine and immune systems, SIRS is also accompanied by hormonal changes. IL-1, IL-6 and TNF-α activate the hypothalamus-pituitary-adrenal axis (HPA) and lead to a steady increase in cortisol production, with subsequent immunosuppression [36,37]. The initial SIRS, which enhances tissue damage, is followed by an anti-inflammatory state meant to restore immune homeostasis and promote wound healing [33].

Our patient experienced multiple trauma during the car accident and underwent major orthopedic surgery, both events leading to a state of systemic immunosuppression that explains the development of DSP. The patient did not present other causes of immunosuppression, solid neoplasms, hematologic malignancies, and viral infections being excluded based on the results of laboratory tests and imagistic investigations.

Koebner phenomenon is another possible mechanism involved in the development of DSP in our patient. Also termed isomorphic response, koebner phenomenon is defined as the appearance of new skin lesions at the site of trauma and is encountered in numerous dermatoses, especially psoriasis, lichen planus, and vitiligo.

Generally, the diagnosis of porokeratosis is established based on the clinical examination given the highly specific aspect of skin lesions and their characteristic distribution. Dermoscopy may prove useful in assisting the clinician to diagnose porokeratosis. The main dermatoscopic feature of porokeratosis is the keratin rim, defined as “a partial or complete, slightly raised, thin, scaly border at the periphery of the lesion with a double free edge” [38]. In DSAP and porokeratosis of Mibelli, other dermoscopic findings include glomerular or dotted vessels, pigmentation or grey-brown dots along the keratin rim, light-brown pigmentation and non-peripheral scales within the keratin rim [38]. In DSP, dermoscopy may reveal brownish pigmentation in the central part of the lesion and a double white track structure at the periphery of the lesion [39].

In less typical cases, the histopathologic examination of a skin biopsy is necessary in order to confirm the diagnosis and exclude other disorders. The skin biopsy should include the peripheral rim of the skin lesion. The histopathological clue for all variants of porokeratosis is the presence of a cornoid lamella, which actually represents a parakeratotic column covering a vertical zone of vacuolated and dyskeratotic cells localized at the epidermic level [40]. The main histopathologic differential diagnoses are porokeratomas, actinic keratoses, psoriasis and warts.

Generally, no laboratory analyses or paraclinical investigations are necessary. Nevertheless, in the setting of severe exacerbations, screening for underlying immunosuppressive conditions is mandatory.

Porokeratosis is considered by some authors to be a premalignant condition [41], the lesions portending a 7.5% risk of degeneration into squamous cell carcinoma [42]. This most commonly occurs in large, long-lasting, or linear cutaneous lesions [42]. Other experts associate it with a general higher risk of developing skin cancer [1].

Depending on the extension of the lesions and the patient’s choice, the therapeutic approach varies widely, from the use of simple emollients and photoprotection to topical immunomodulatory treatments (5-fluorouracil, imiquimod, vitamin D3 analogues, or calcineurin inhibitors), topical anti-inflamatory products (diclofenac 3% gel), and topical or systemic retinoids. The latter, apart from normalizing keratinocyte differentiation, also prevent malignant degeneration in high-risk porokeratosis patients.

Various minimally invasive procedures may be employed with good cosmetic results, such as curettage, cryotherapy, electrodessication, and laser therapy. Photodynamic therapy has also been successfully used for the treatment of DSAP and linear forms of disease [42].

However, in most cases, the approach is limited to reassurance of the patient and long-term follow-up in order to promptly detect potential malignant transformation within the skin lesions.

In the absence of specific treatment, porokeratosis lesions are usually long-lasting, showing no tendency of spontaneous remission. In our patient, the lesions resolved spontaneously few months after their occurrence. We hypothesize that, as the patient’s health improved significantly and the patient regained immune competence, having no persistent risk factor for the development of porokeratosis, the disease activity subsided and eventually entered remission. 

## Conclusions

To our knowledge, this is the first case of DSP occurring as a consequence of post-traumatic immunosuppression reported in the medical literature. Spontaneous complete remission of DSP may occur upon restoration of immune competence in patients with no other predisposing factors. Therefore, the approach in such cases may be limited to reassurance of the patient and careful monitoring.
